# Distinct benthic and planktonic microbial communities within the stratified water column of the perennially ice-covered Lake Joyce, Antarctica

**DOI:** 10.1093/femsec/fiag078

**Published:** 2026-08-06

**Authors:** Carla Greco, Ian Hawes, Dawn Y Sumner, Tyler J Mackey, Gonzalo Oteo-García, Marian Yallop, Gary Barker, Rachael Morgan-Kiss, Anne D Jungblut

**Affiliations:** Natural History Museum, Department of Sciences, London, SW7 5BD, United Kingdom; School of Biological Sciences, University of Bristol, Bristol, BS8 1TQ, United Kingdom; Coastal Marine Field Station, University of Waikato, Sulphur Point, Tauranga 3110, New Zealand; Department of Earth and Planetary Sciences, University of California Davis, Davis, One Shields Avenue, Davis, CA 95616, United States; Department of Earth and Planetary Sciences, University of New Mexico, NM 87131, United States; Centre for Palaeogenetics, Stockholm University, Frescativägen 8, SE-114 18 Stockholm, Sweden; Dipartimento di Biologia Ambientale, Sapienza Università di Roma, Via Cesare de Lollis, 21, 00185 Rome, Italy; Department of Archaeology and Classical Studies, Stockholm University, Wallenberglaboratoriet, SE-106 91 Stockholm, Sweden; School of Biological Sciences, University of Bristol, Bristol, BS8 1TQ, United Kingdom; School of Biological Sciences, University of Bristol, Bristol, BS8 1TQ, United Kingdom; Department of Microbiology, Miami University, Oxford, OH 45056, United States; Natural History Museum, Department of Sciences, London, SW7 5BD, United Kingdom

**Keywords:** 16S rRNA gene, 18S rRNA gene, microbial mat, microplankton, lake, Antarctica

## Abstract

Perennially ice-covered lakes are hotspots of biodiversity and biomass accumulation in polar deserts. Many Antarctic perennially ice-covered lakes are density stratified, within which an oxygenated upper layer overlies deeper, darker anoxic waters. We carried out the first 16S and 18S rRNA gene high throughput sequencing of microplankton and benthic microbial mats in perennially ice-covered Lake Joyce, McMurdo Dry Valleys, Antarctica. We evaluated community composition and richness at different lake and water depths, and determined the level of connectivity of benthic and planktonic microbial communities. While some overlap between benthos and plankton was observed, the microbial communities were distinct in richness and community composition. The benthic communities comprised microbial mats, which were dominated by Cyanobacteria and Proteobacteria, whereas the planktonic community showed an increased contribution of Bacteroidota. A higher number of 16S rRNA gene ASVs were specific for the plankton than the mats, whereas more 18S rRNA gene ASVs were specific to the microbial mats than the plankton. The benthic eukaryotic community was dominated by ciliates and diatoms while Cryptophyceae were abundant in the plankton. These findings demonstrate that both benthic and planktonic communities need to be considered for census of taxonomic richness, productivity, and ecological processes occurring within Antarctic lakes.

## Teaser

Ice-covered Lake Joyce hosts stratified and distinct benthic microbial mat and planktonic microbial communities underscoring the role of both habitats in Antarctic lake ecology.

## Introduction

The McMurdo Dry Valleys (MDV) region is a cold and dry polar desert that spans about 4500 km^2^, and the largest ice-free region of Antarctica (Levy 2013) includes a group of perennially ice-covered, closed-basin, meromictic lakes with long, complex histories that have been studied extensively in the last decades (Doran et al. 1994, Green and Lyons 2009). These lakes are extreme environments, characterized by persistent low temperatures, irradiance, and oligotrophic conditions. Despite this, they are hotspots of biodiversity in polar deserts, supporting high biodiversity, biomass accumulation, and productivity both in the benthos (Quesada et al. 2008) and the plankton (Priscu 1995). Consistent features of both communities include truncated food webs, with low abundance of micro- and meso-fauna, and dominance by microbial communities. The dominant benthic communities within the photic zones of these lakes comprised laminated cyanobacteria-based microbial mats, structured by low disturbance without macroscopic bioturbation and grazing (Hawes et al. 2019). These microbial mats can have a thickness of several centimeters and consistently provide higher biomass stocks than microplankton under the prevailing oligotrophic water conditions (Hawes et al. 2001). In contrast, eukaryotes are often dominant planktonic primary producers, with a range of photo- and mixotrophic flagellates tending to dominate, and heterotopic and predatory protists are also common (Morgan-Kiss et al. 2024). Environmental conditions play important roles in assembly of prokaryotes in microbial mats and bacterioplankton communities in Antarctic aquatic ecosystems such as the Wright Valley, McMurdo Dry Valleys (Ramoneda et al. 2021). It has been shown for Arctic lakes that microplankton growth is limited by nutrients whereas microbial mats are able to retain and recycle nutrients within the mat habitat (Bonilla et al. 2005), and the same has been shown in Antarctica (Priscu 1995, Quesada et al 2008).

Surveys of 16S and 18S rRNA genes within microbial communities in McMurdo Dry Valley Lakes Bonney, Vanda and Fryxell, have added to the understanding of biodiversity and environmental drivers, but to date have focused solely on either benthic microbial mats or the planktonic communities. They therefore provide little information on the similarities and differences in community composition and richness between these different habitats, particularly with respect to microbial eukaryotes. As such, it has been difficult to create a direct comparison of the response of both communities to environmental conditions along physical and chemical gradients. Additionally, long-standing questions remain regarding habitat specificity of the microbial taxa identified from microbial mats versus microplankton in the water column, the level of habitat coupling and movement of organisms between benthic environments and their overlying waters.

Our study is focused on the perennially ice-covered Lake Joyce (77°43.2′S, 161°37.2′E). It is a proglacial, meromictic lake located in the Pearse Valley of the MDV in Southern Victoria Land, Antarctica, and the Taylor Glacier forms the south-east boundary of the lake. Lake Joyce is the least investigated lake of the MDVs and has some unique geochemical and biological features in comparison to the relatively better-studied McMurdo Dry Valley lakes including Lakes Bonney, Vanda and Fryxell. These differences include inorganic carbon chemistry, with high pH (approaching pH 10), and supersaturation in calcite in surface waters. Consequentially there are three dimensional microbial structures formed by the microbial mats, with carbonate skeleton (Mackey et al 2017). In addition, Lake Joyce is unique in possessing a substantial population of the copepod *Diacyclops joycei*, which has nauplius larvae that are planktonic and adults that associate with benthic mats (Karanovic et al. 2014, Mackey et al. 2026). In this study, we investigated for the first time the microbial community structure and richness covering Bacteria, Archaea, and microbial eukaryotes in the water column and the benthic microbial mats along the bottom of Lake Joyce at different lake depths using high-throughput 16S and 18S rRNA gene sequencing. The study includes two morphologies of microbial mats, as the benthos contain flat prostrate microbial mats and three-dimensional pinnacles (Hawes et al. 2011). We hypothesized that the enhanced microhabitat variability within laminated microbial mat communities would result in a higher microbial richness, but that differences in community composition would be higher amongst planktonic populations structured by the stratification of the water column. This is relevant to resolve drivers of community structure and understand the contribution of both benthic and planktonic environments to biodiversity in freshwater environments in polar deserts.

## Material and methods

### Study Site

**Figure 1:**
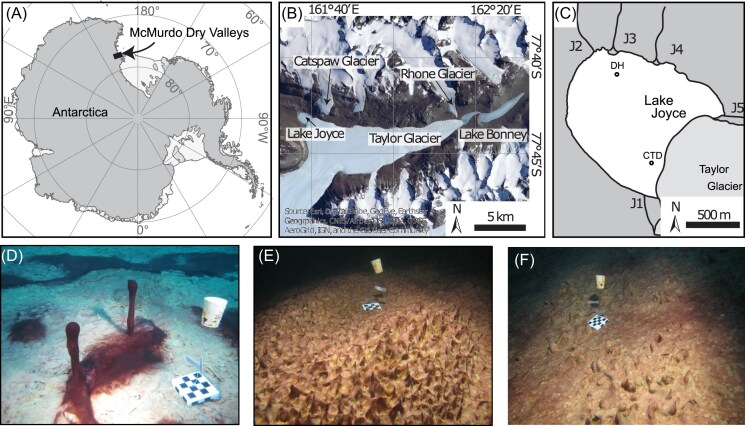
Geographic location and photographs of diverse microbial mat pinnacles in Lake Joyce, McMurdo Dry Valleys. Antarctica: (A) Location of the McMurdo Dry Valleys. (B) Location of Lake Joyce within the McMurdo Dry Valleys. (C) Schematic of Lake Joyce showing sampling site locations. Location of delta stream (J3) is indicated in the schematic. Microbial mat sampling dive hole (DH) and the sampling hole for water samples and CTD deployment (CTD) are labelled in the schematic. Image modified from Mackey et al. (2017). (D) large finger pinnacle formed lifting due to bubble formation in the cyanobacterial mat (C) webbed pinnacles (F) delta pinnacles—isolated unwebbed pinnacles from site close to delta stream. Squares on float are 1 cm^2^. Photographs taken by Tyler Mackey and Ian Hawes.

Lake Joyce (77°43.2′S, 161°37.2′E) is a perennially ice-covered, proglacial, meromictic lake located in the Pearse Valley of the MDV in Southern Victoria Land, Antarctica (Fig. 1). The surface of Lake Joyce is ∼1 km² and is covered by a 4–7 m thick ice cover that is bounded by a meltwater moat during the summer period. The ice cover transmits 1.7± 0.7% incident photosynthetically active radiation (PAR) (Hawes et al. 2011). Lake Joyce has several meltwater inputs from the adjacent Taylor Glacier and surrounding alpine glacier streams (Fig. 1) but has no evident outflow (Green et al. 1988).

The mean annual temperature of the MDV is approximately −18°C (Doran et al. 2002), meaning that meltwater production occurs mostly during the austral summer periods when warm air temperatures or direct solar heating causes ice melt. In recent years the inflow of meltwater has exceeded the loss to evaporation and ablation, resulting in a gradual increase in the level of the lake (Hawes et al 2011). Annual meltwater influx brings mud-sized grains into the water column of Lake Joyce, which settles onto the lake floor (Squyres et al. 1991). Additionally, sediment can also be composed of wind-blown sand-sized grains that melt through the ice-cover in isolated sedimentation events (Lancaster and Lancaster 2002, Riviera-Hernandez et al. 2018). Sedimentation varies in different sections of the lake with higher sedimentation rates found near streams infloces and decreasing along the shorelines away from these streams (Mackey et al 2017, Fig. 1).

The geochemistry of Lake Joyce has been reported by Shacat et al. (2004). The water column is density stratified due to increasing salinity with depth, with fresh, hyperoxic, high pH water (up to pH 10) overlaying an anoxic layer of higher salinity and lower pH. The upper waters are low in phosphate (0.05 μmol l^−1^), but relatively rich in inorganic nitrogen (26 μmol l^−1^ nitrate-N and 0.1 to 0.2 μmol l^−1^ nitrite-N). Green et al. (1988) reported dissolved iron below detection limits of 13 nmol l^−1^ in surface waters, increasing dramatically in the anoxic zone. Shacat et al. (2004) did not report quantitative hydrogen sulphide data, however, they did report that its presence was detected by odor from below 27 m in the water column. Green et al. (1988) reported that Lake Joyce was the most calcite supersaturated lake in the MDV, particularly in the upper part of the water column.

The floor of Lake Joyce is covered by benthic microbial mats to a depth of ∼23 m (Hawes et al. 2011), largely composed of mat-forming filamentous Cyanobacteria. The microbial mat forms distinct morphologies, from flat prostrate mat to calcified microbialites (sensu Mackey et al 2015, 2017). Mackey et al. (2015), Grey and Awramik 2020 reported several different microbial mat morphologies in addition to flat prostrate mat, including buoyantly supported dm-scale, unlithified columnar structures without branching, termed “finger pinnacles” (Fig. 1), at depths shallower than 10 m (Mackey et al. 2018). There were also two types of pinnacles reported at depths up to 15 m, specifically “webbed pinnacles” which have delicate features including vertical webs of 10–100 μm thick biofilms suspended between pinnacles (Fig. 1), and “isolated pinnacles” lacking the delicate connecting webs (Fig. 1F) Both morphologies are typically lithified with subsurface calcite covered by a layer of microbial mat (Hawes et al. 2011, Mackey et al. 2015). Observations by Mackey et al. (2017) showed a transition in the abundance of different pinnacle morphologies from isolated to webbed that correlated with higher to lower sedimentation regions of the lake.

The microbial communities of Lake Joyce have previously been characterized through microscopy-based methods. Benthic communities were dominated by Cyanobacteria *Pseudanabaena frigida, Leptolyngbya spp*., and *Phormidium autumnale*, and planktonic communities by phototrophic nanoflagelletes, filamentous Cyanobacteria, and the ciliate Plagiocampa (Roberts et al. 2004, Hawes et al. 2011, Mackey et al. 2017). Roberts et al. (2004) also reported a number of copepod nauplii in plankton samples, and epibenthic adult copepods were first described in the lake by Karanovich et al. (2014).

### Sample collection and environmental measurements

Measurements of temperature, specific conductivity, dissolved oxygen, chlorophyll-*a*, and phycocyanin in the water column were made in November to December 2014 through a hole in the lake ice cover at the deepest known part of Lake Joyce (Fig. 1) using a YSI 6600 Sonde.

The benthic microbial mats and plankton in Lake Joyce were sampled between late November and early December in 2014. Microbial mats only occurred in the photic zone and were collected by divers by cutting out samples from *in situ* mats using a spatula and transferring them into sterile plastic containers. Due to logistical constraints benthic samples could only be collected from five lake depths. A total of 42 benthic mat samples were collected from 7.4, 9.6, 12.0, 13.4, and 17.4 mat two different locations accessed through one dive hole (Table 1; Mackey et al. 2017). The sites were different distances from a meltwater stream described by Mackey et al. (2017). “Delta” samples (Abbreviated: PD) were collected at the site closer to stream J3, and “non-delta” samples (Abbreviated: P) were collected farther from the stream mouth (Fig. 1). Prostrate mats were collected in triplicate at 9.6 m and 12.0 m depth, and at 7.4 m and 17.4 m at the non-delta site. In addition to prostrate mats, triplicate samples were collected from three different mat morphologies: finger pinnacles, webbed pinnacles, and isolated pinnacles (Fig. 1) that represented morphologies present across the lake bottom at this site. Biomass for the three webbed (Abbreviated as WU for webbed upper layer, and WL for webbed lower layer) and three isolated pinnacles (“drum shaped”) were collected to have triplicate samples per type of pinnacle (Abbreviated as DU for upper layer, and DL for lower layer). The pinnacles were dissected into upper purple-green pigmented surface layer and lower beige coloured layer immediately upon transfer to the field laboratory using sterile equipment. A single large finger pinnacle was collected at 7.4 m. Due to field work limitations, only one large finger pinnacle could be collected; it was used to provide three technical replicates of each the upper (FU) and lower (FP) layers of the mat.

**Table 1:** Sample codes, description, and locations of benthic and planktonic samples from Lake Joyce. Location of dive hole (DH) and CTD sampling holes and delta stream are shown in Fig. 1.

Sample	Description	Depth (m)	Location
P	Prostrate	7.4, 9.6, 12, 17.4	DH (far from delta)
PD	Prostrate delta	9.6, 12	DH (near delta)
FU	Finger pinnacle upper layer	7.4	DH
FL	Finger pinnacle lower layer	7.4	DH
DU	Isolated pinnacle upper layer	12	DH (near delta)
DL	Isolated pinnacle lower layer	12	DH (near delta)
WU	Webbed pinnacle upper layer	13.4	DH (far from delta)
WL	Webbed pinnacle lower layer	13.4	DH (far from delta)
WTR	Water column	12, 18, 22, 25, 27, 37	CTD

For the planktonic community, water samples (Abbreviated: WTR) were collected using a Kemmerer water sampler from lake water depth of 12, 18, 22, 25, 27, and 37 m through a hole in the ice of at the deepest point of Lake Joyce. These depths were selected on the basis of the water column profile obtained prior to water sampling. Samples were transferred into cubitainers that had been pre-washed with 10% HCl washed, and rinsed with sterile deionized water and then three times with sample water. Biomass was collected from the water samples by filtering 1.9–3.7 l through sterile Sterivex-GP R filter units (Merck Millipore, Darmstadt, Germany) with 0.22 μm pore size using a Masterflex peristaltic pump system. The biomass was stored in a lysis buffer at −20°C immediately after processing. All samples were stored frozen for the remainder of the field season, shipped at −20°C, and stored at −80°C until DNA extraction at the Natural History Museum, London, UK.

Abundance of major algal classes were quantified as Chl*a* fluorescence across the water column in Lake Joyce during the field season 2013–2014 using a submersible spectral fluorometer (FluoroProbe, BBE Moldaenke) (Patriarche et al. 2021).

### DNA extraction, polymerase chain reaction (PCR), and sequencing

DNA was extracted from between 0.05 and 0.3 g of microbial mat biomass using the MoBio PowerBiofilm DNA Extraction kit (Carlsbad, CA) following the manufacturer’s instructions. The DNA of planktonic communities was extracted from biomass concentrated on Sterivex filters using a salt (NaCl)-base protocol modified from Aljanabi and Martinez (1997) with lysozyme and proteinase K (Diez et al. 2001) for Arctic and Antarctic freshwater environments as detailed by Ramoneda et al. (2021) and Charvet et al. (2014). The 16S rRNA gene was amplified in triplicate using 515F-806R primers (Caporaso et al. 2012) and the 18S rRNA gene was amplified in triplicate using the 1391f-EukBr primers (Amaral-Zettler et al. 2009) as described in Greco et al. (2020). The PCR reaction volume was 20 μl with 0.2 μl of 5 μgμl−1 GoTaq polymerase, 4 μl 5X GoTaq Flexi buffer, 8.84 μl sterile PCR-grade water, 0.8 μl of 20 mgml−1 bovine serum albumin (BPS), 2 μl of 24 μmoll−1 magnesium chloride (MgCl2), and 0.16 μl of 200 μmoll−1 dNTPS (deoxynucleotides triphosphate). Samples were then amplified using a thermal cycler using the following programs. For the 16S rRNA gene the program was as follows: 94C for 3 min, 35 cycles of: 94°C for 45 s, 50°C for 60 s and 72°C for 90 s, followed by a final 7°C step for 10 min. For the 18S rRNA gene a program of 94C for 3 min, 35 cycles of: 94°C 45 s, 57°C for 60 s, and 72°C for 90 s, followed by a 72°C for 10 min was used. The amplified PCR-products were visualized on 1% agarose gel using electrophoresis at 100 V for 30 min. The 16S rRNA and 18S rRNA gene PCR products were purified using AxyPrep Mag PCR clean-up (Axygen, New York, NY, United States), and quantified using a Qubit fluorometer (Invitrogen, United Kingdom) and pooled prior to sequencing. The multiplexed amplicons were sequenced on an Illumina MiSeq platform at the Natural History Museum Molecular Laboratories. The 16S and 18S rRNA gene sequencing data has been submitted to the Short Read Archive in GenBank under the Bioproject PRJNA1417219.

### Bioinformatic and statistical analysis

Sequences were processed in QIIME2 (version 2018.8) (Bolyen et al. 2019) using recommendations from the standard operating procedures from the Microbiome Helper repository (Comeau et al. 2017). Sequences were quality filtered, merged, and the DADA2 algorithm (Callahan et al. 2019, 2016) was applied to denoise sequences and generate an Amplicon Sequence Variant (ASV) table. Taxonomy was assigned using the q2-feature-classifier (Bokulich et al. 2018) classify sklearn naive Bayes taxonomy classifier against the SILVA 138 database (Quast et al. 2013, Yilmaz et al. 2014). The SILVA 138 database was formatted using the RESCRIPt QIIME 2 plugin (Robeson II et al. 2021). All mitochondrial and chloroplast plastid 16S rRNA gene sequences based on this classification were removed from the 16S rRNA gene data set, and any bacterial sequences amplified due to unspecific binding of the primers were removed from the 18S rRNA data set. Low abundance ASVs were filtered using a threshold of < 0.1% of mean sequencing depth, as specified in the Microbiome Helper repository (Comeau et al. 2017).

Statistical analyses were conducted in R version 3.5.2 using packages using phyloseq (version 1.26.1) (McMurdie and Holmes 2013) and vegan (version 2.5–6) (Oksanen et al. 2019). Alpha diversity was tested using Observed ASVs, Shannon’s and InvSimpson Indexes, and differences between diversity indices were tested with a Mann–Whitney U test or a Kruskal–Wallace test followed by a Dunn’s test as a post hoc test (Bonferrroni corrected). Principal Coordinate Analysis (PCoA) was performed using Bray–Curtis dissimilarity on the (relative abundance transformed) ASV table. Significant differences in beta diversity between sample groups were determined using Permutational Multiple Analysis of Variance (PERMANOVA) in R using the adonis function. Hierarchical clustering was computed using Bray–Curtis dissimilarity with a UPGMA clustering method. Differential abundance of taxa (summarized to genus level) between groups were calculated using the DESeq2 R package (version 1.22.2) with an adjusted *P*-value threshold of 0.01 (Love et al. 2014). Due to the inconsistency of comparing “bulk” communities to dissected layers, the dissected pinnacles were not directly compared to the flat (undissected) samples and the water column samples, and were investigated independently.

### Evolutionary placement analysis

A reference cyanobacterial tree was constructed that includes the closest matches to the SILVA database for each cyanobacterial ASV. Additionally, cyanobacterial 16S rRNA gene sequences from published Arctic and Antarctic studies and reference strains of the identified morphotypes were also included in the phylogenetic tree for comparison. The reference sequences were aligned using ClustalX (version 2.1) (Larkin et al. 2007) to produce a 640 bp multiple sequence alignment, and a reference maximum-likelihood (ML) phylogenetic tree was constructed in RaxML (version 8.2.12) (Stamatakis et al. 2008) using the GTRCAT substitution model for the bootstrapping phase and the GTRGAMMA for the final tree inference. A best scoring ML tree was obtained based on 1000 bootstraps. The cyanobacterial ASVs obtained in this study were aligned using PaPaRa 2.5 (Berger and Stamatakis 2012) and mapped onto the reference tree using the Evolutionary Placement Algorithm (EPA-NG) (Barbera et al. 2019) and only read placements with likelihood weight of >0.5 were retained.

## Results

### Water column profile

The water column was stratified, with a near isohaline, low conductivity upper layer overlying a distinct chemocline that began at 16 m depth and reached a maximum rate of conductivity change between 20 and 25 m (Supplementary Figure 1). Below the steep chemocline, the conductivity of the water continued to increase gradually. Temperature varied only by 0.5°C through the profile, being close to the freezing point in the upper layer, rising slightly at the chemocline then falling to below zero in the saline deep water. The upper waters had a pH exceeding 10 in places, however the pH declined to between 7 and 8 at the chemocline. The highest concentration of DO was 35 mg l^−1^ in the upper water column, DO began to decline at around 21 m, and the water was anoxic below 27 m.

Community composition and distribution of four major spectral algal classes (green algae, cyanobacteria, mixed, and cryptophytes) were determined in Lake Joyce using *in situ* Chl-*a* fluorescence measurements indicated Cryptophyte and mixed communities to have the highest concentrations of the four classes with peaks inconcentrations between 9 and 20 m lake depths. Green algae Chl-*a* were detected across the water profile with highest concentrations in upper water and decreasing concentrations with increasing lake depth. Cyanobacteria Chl-*a* was in comparison only measured at low concentrations across the water profile (Supplementary Figure 2).

### rRNA gene and 18S rRNA gene microbial composition in Lake Joyce

**Figure 2:**
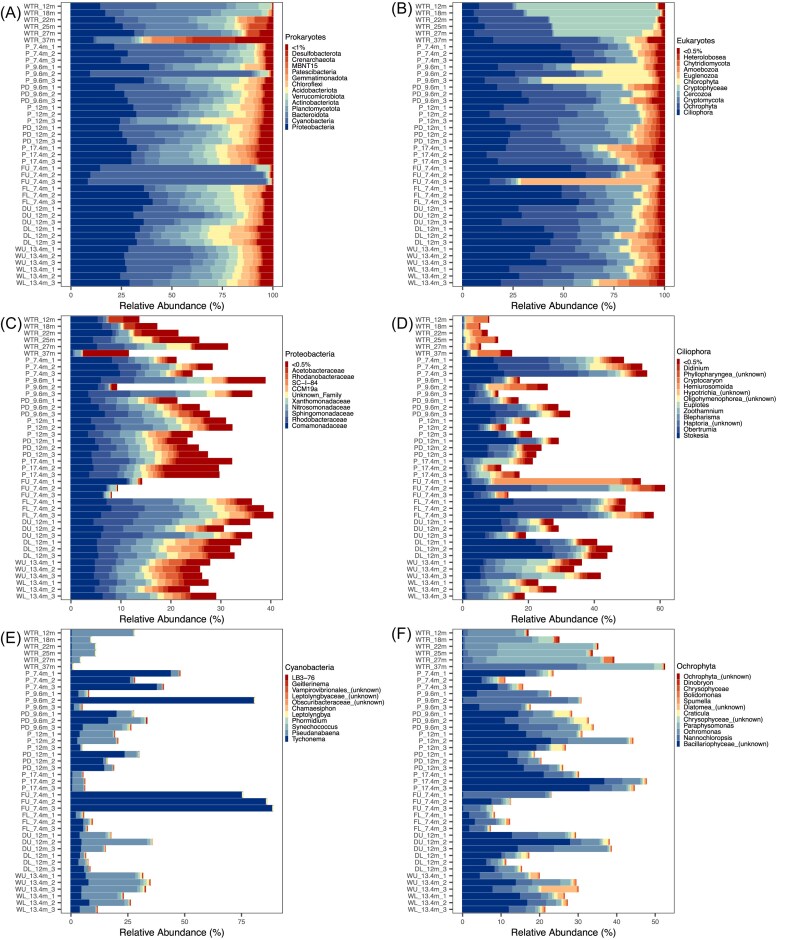
Relative abundance plots of (**A)** bacteria and archaea (**B)** eukaryotes (**C)** Proteobacteria at family level (**D)** Ciliophora at genus level (**E)** Cyanobacteria at genus level (**F)** Ochrophyta at genus level in planktonic, bulk flat mats, and dissected microbial pinnacles. Taxa that have a mean abundance below a certain threshold (n) are labelled as <n%. Taxa that could not be identified at family or genus level are classified at their highest taxonomic assignment and labelled “(unknown)”. Abbreviations: WTR, water column; P, prostrate mat; PD, prostrate mat nearer to delta; FU, upper layer of finger shaped pinnacle, FL, lower layer of finger shaped pinnacle, DU upper layer of isolated pinnacle; DL, lower layer of isolated pinnacle; WU, upper layer of webbed pinnacles; WL, lower layer of webbed pinnacle.

For the 16S rRNA gene dataset 4 995 590 sequences and 1205 ASVs were identified across benthic microbial mat and planktonic microplankton communities. The most abundant phyla were Proteobacteria (26.8%), Cyanobacteria (24.0%), Bacteroidota (9.8%), Planctomycetota (8.1%), Actinobacteriota (7.0%), Verrucomicrobiota (5.7%), Acidobacteriota (4.1%), and Chloroflexi (4.0%) (Fig. 2). For Proteobacteria, the most abundant genera were *Polaromonas* (2.2%), Ellin6067 (1.91%), *Sphingopyxis* (1.4%), and *Rhodoferax* (1.4%). The most prevalent Proteobacteria orders were: Burkholderiales (9.8%), Rhodobacterales (3.9%), Sphingomonadales (3.08%), Xanthomonadales (2.8%), Gammaproteobacteria_Incertae_Sedis (1.4%). In addition, three archaeal ASVs were identified, assigned as Crenarchaeota Candidatus *Nitrosopumilus* and Nanoarchaeota Woesearchaeales.

The cyanobacterial community was largely comprised of the genera *Tychonema* (14.8%), *Pseudanabaena* (8.2%), *Synechococcus* (0.45%), *Phormidium* (0.3%), and *Leptolyngbya* (0.2%). Phylogenetic placement analysis of the 19 cyanobacterial ASVs confirmed the taxonomic assignment by SILVA to cyanobacterial genera for most ASVs (Supplementary Figure 3). A potential exception is the *Synechococcus* ASVs identified which were classed as *Synechococcus* PCC-7502, a Pseudanabaenales related to *Pseudanabaena* PCC7403. Sister clades to cyanobacteria Obscuribacterales (1 ASV) and Vampirovibrionales (2 ASVs) were identified in the samples at low abundance. Several ASVs were related to environmental sequences and cyanobacteria strains previously identified from microbial mats in Antarctica; two ASVs mapped to sequences from MDV Lake Hoare and five ASVs to MDV Lake Vanda. *Chamaesiphon* mapped to environmental sequences from Dry Valley Lake Hoare, *Phormidium, Pseudanabaena*, and *Synechoccocus* to Dry Valley Lake Vanda, and Leptolyngbyacea ASVs mapped to both Lake Hoare and Lake Vanda sequences.

**Figure 3:**
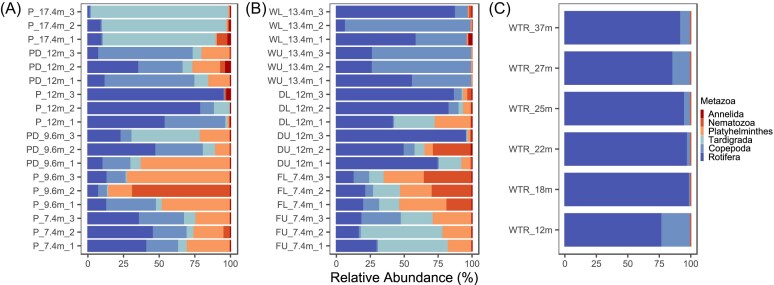
Relative abundance of metazoan phyla in (A) flat prostrate mat (B) finger, webbed pinnacles, and isolated pinnacles; and (C) planktonic samples. Abbreviations: WTR, water column; P, prostrate mat; PD, prostrate mat nearer to delta; FU, upper layer of finger shaped pinnacle, FL, lower layer of finger shaped pinnacle, DU upper layer of isolated pinnacle; DL, lower layer of isolated pinnacle; WU, upper layer of webbed pinnacles; WL, lower layer of webbed pinnacle.

In the 18S rRNA gene dataset 1 346 651 sequences from 425 ASVs were identified, 18 of which were metazoa. The most abundant groups identified were Ciliophora (28.4%), Ochrophyta (25.8%), Cryptomycota (9.6%), Cercozoa (9.1%), Cryptophyceae (7.2%), and Chlorophyta (5.7%) (Fig. 2). The most abundant Ciliophora genera were *Stokesia* (7.4%), *Obertrumia* (4%), and *Haptoria* (unknown genus) (3.8%). The most abundant Cercozoa were: Vampyrellidae (unknown genus) (3.4%), *Spongospora* (0.8%) and *Euglyphida* (unknown genus) (0.8%). Within Ochrophyta, Diatomea (11.5%), Chrysophyceae (8.3%), and Eustigmatophyceae (5.9%) formed the most common groups. Diatoms were largely composed of ASVs from an unknown genus of order Bacillariophyceae (10.4%). The most abundant Chrysophyceae was *Ochromonas* (4.5%) and Eustigmatophyceae was *Nannochloropsis* (5.9%). The most abundant Cryptophyceae ASV was not assigned a genus by SILVA (closest match to Cryptophyta sp. SL64/78sp), however, was found to have a 100% BLAST match to a strain of *Geminigera cryophila* strain from Bayly Bay, Antarctica. Metazoa had a patchy distribution and high variability between the mat samples and were largely composed of rotifers (Bdelloidea and Monogononta) (Fig. 3). A copepod of order Cyclopoida was also identified in a number of samples.

### Bacteria, archaea, and microbial eukaryote planktonic communities across the stratified water column of Lake Joyce

The water column prokaryote biota was largely composed of Bacteroidota (mean 31.2%), Proteobacteria (mean 20.4%), and Cyanobacteria (mean 10.5%) (Fig. 2). The most abundant eukaryote community groups were Cryptophyceae (mean 37.7%), Ochrophyta (mean 33.7%), and Ciliophora (mean 8.6%). The cyanobacterial community in the water column had a low diversity, with *Pseudanabaena* PCC-7429 contributing 99.4% of the planktonic cyanobacteria ASVs.

The planktonic 16S and 18S rRNA gene communities showed a shift along the stratification of the lake conditions as shown in Supplementary Figure 1. Particularly in the relation to oxygenation. In the 16S rRNA dataset, a decrease in Cyanobacteria was observed with depth, from 27% in the 12 m sample (euphotic zone), to 4.1% at 27 m, towards the base of the oxycline and 0.8% in anoxic water at 37 m.

In the shallow and oxygenated water (12 m and 18 m), the community was composed mostly of Bacteroidota, Cyanobacteria, Proteobacteria, and Actinobacteria (Fig. 2). In the transition to the euxinic zone, an increase in relative abundance of Crenarchaeota—Candidatus *Nitrosopumilus*—was observed (6.8% at 22 m, 8.7% at 25 m, and 6% at 27 m). In the anoxic zone in the 37 m sample an increase in Desulfobacterota (21.7%), Patescibacteria (11.4%), Firmicutes (6.5%), MBNT15 (6%), and Campilobacterota (5.8%) was observed, whereas in the shallow water where they were observed at extremely low proportions (0%–0.2% in the shallow 12 m and 18 m samples). Abundant Desulfobacterota were classified as *Desulfurivibrio, Smithella*, and *Desulfobacca*. Within Patescibacteria, the most abundant ASVs were classified as Candidatus *Moranbacteria* and *Campilobacterota* was identified as *Sulfuricurvum*. A decrease in Bacteroidota, Proteobacteria, and Actinobacteriota was observed at 37 m (17.9, 11.5, and 2.5%, respectively). *Gallionella* (iron-oxidizing Proteobacteria) increased in abundance in the anoxic zone from 0% to 0.1% in 12–27 m, to 5.6% at 37 m, comprising the most abundant Proteobacteria at the deepest depth. Similarly, strictly anaerobic *Lentimicrobium* (Bacteroidota) increased from 0% in the oxic (12–27 m) to 4.9% in the 37 m sample.

In the 18S rRNA gene community, a decreasing trend in Cryptophyceae was observed with a sharp decrease in relative abundance at 37 m (from 69.6% at 12 m to 6.8% at 37 m, Fig. 2). An opposing trend was observed with Ochrophyta, from 17% to 52.4% in 12 m and 37 m, respectively. Within Ochrophyta a shift was observed; *Paraphysomonas* increased in relative abundance after 22 m (from 2.2% and 1.3% at 12 m and 18 m, respectively, to 25% at 22 m), and mixotrophic *Nannochloropsis* increased in the anoxic waters from 1.2% to 2.5% in the 12 to 27 m samples to 30% at 37 m. Conversely, the mixotrophic *Ochromonas* decreased from 12.7% at 12 m to 2.1% at 37 m.

### Prostrate microbial mat communities with lake depth

**Figure 4:**
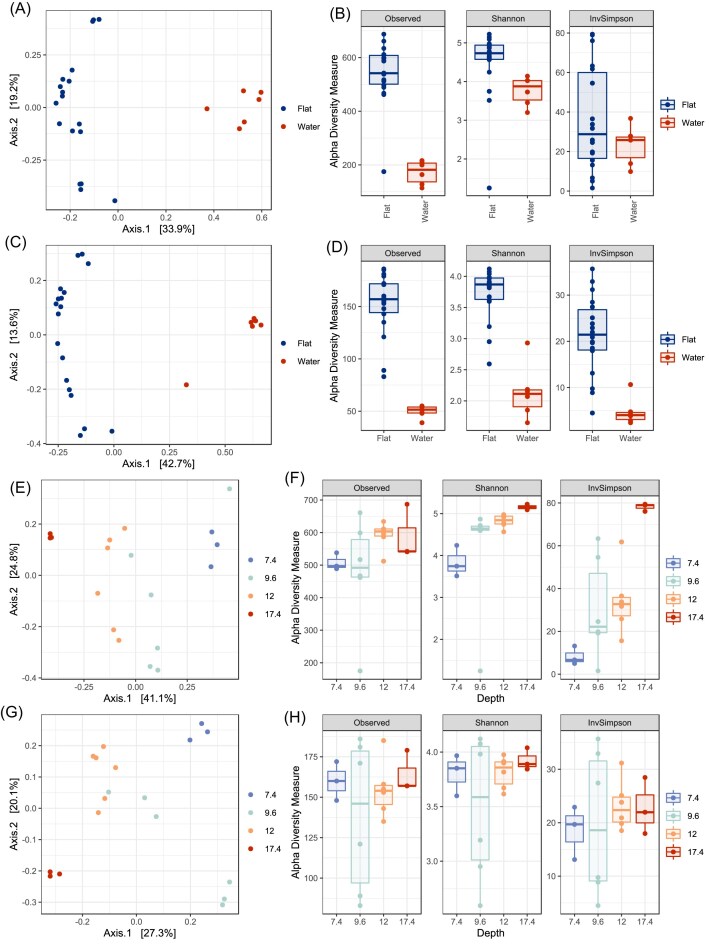
Principal coordinate analysis (PCoA) ordination patterns based on Bray–Curtis dissimilarity index of prostrate mat and water column samples for (A) 16S and (C) 18S rRNA gene dataset, and for the prostrate flat mats along a depth gradient for the (E) 16S rRNA and (G) 18S rRNA dataset. Alpha diversity indices Observed, Shannon, and InvSimpson for prostrate mat and water column samples (B) 16S rRNA and (D) 18S rRNA datasets, and along a depth gradient for the prostrate mats for the (F) 16S rRNA and (H) 18S rRNA datasets.

Prostrate mats were collected as bulk material, containing multiple layers from highly pigmented to low in pigmentation. The most abundant phyla in the flat mat communities were Proteobacteria (mean 27.4%) and Cyanobacteria (mean 23.5%), Ochrophyta (mean 28.1%), and Ciliophora (mean 26.1%) (Fig. 2 A–D). At phylum level a change in composition could be observed with depth in the prostrate mat. A gradual decrease relative abundance of Cyanobacteria was observed from 7.4 m (mean 38.8%), 9.6 m (non-delta samples mean 31.1%, delta samples mean 29%), 12 m (non-delta samples mean 14.91% and delta samples mean 21.6%), to 17.4 m (mean 5.8%). *Tychonema* was most affected by depth, decreasing from mean 37.1% in the 7.4 m samples to 0.3% in the 17.4 m samples. *Pseudanabaena* showed less variability with depth, and reached peak abundance in the PD 9 m samples (mean 12.8%). Additionally, a trend of increasing diversity with depth was observed in the 16S rRNA gene dataset for the Shannon and InvSimpson indices, with a significant difference between 7.4 m and 17.4 m samples (Shannon *P* < 0.01, InvSimpson *P* < 0.01) which was not observed for the 18S rRNA gene (Fig. 4B and D). No clear differences were observed for the 16S and 18S rRNA gene mat communities from the two sampling locations at the same lake depth (Fig. 4F and H).

PCoA analysis of 16S rRNA gene communities showed that communities tended to cluster by lake depth based on the available number of samples. In particular, the mats assemblage samples from 7.4 m and 17 m tended to cluster more closely with samples from the same depth and separately from each other (Fig. 4E and G), which was also observed for the 18S rRNA gene communities. This clustering pattern was supported by a significant PERMANOVA result (16S rRNA gene: R^2^ = 0.53, F = 7.08, *P* < 0.01; 18S rRNA gene: R^2^ = 0.53, F = 5.25, *P* < 0.01). However, low sample numbers meant no pairwise adonis analyses could be undertaken.

### Microbial communities in pinnacles

Microbial finger, webbed and isolated pinnacles were analyzed as dissected layers, composed of upper pigmented layers and lower non-pigmented layers. At phylum level, a clear distinction can be seen between the FU and FL samples: Cyanobacteria increased in FU (mean 83.44%) and decreased in FL (mean 7.39%), whereas Proteobacteria decreased in FU (mean 10.50%) and increased in FL (mean 33.10%) (Fig. 2A). The distinct community of the 16S rRNA FU samples was supported by hierarchical clustering where the three FU formed a distinct clade (Supplementary Figure 4). The lower and upper layer of the webbed pinnacles (WU and WL) formed a sub clade with the lower isolated pinnacles (DL) as a sister clade. Another further clade contained the upper layer of isolated pinnacles (DU) and the lower layer of the finger pinnacle (FL). Differentially abundant taxa that contributed to this clustering, tested through the DESEq2 algorithm, are listed in Supplementary Table 2.

### Comparison of prostrate microbial mat and microplankton communities

Prostrate microbial mat and water habitats were compared and contained distinct bacterial and eukaryote assemblages, with clear clustering evident (Fig. 4A and C, 16S rRNA gene dataset, PERMANOVA: F = 10.52, R2 = 0.32, *P* < 0.01, and 18S rRNA gene dataset (Fig. 4B and D, PERMANOVA: F = 15.015, R2 = 0.41, *P* < 0.01). There were 208 significantly different prokaryotic taxa (genera or highest taxonomic assignment) between water and bulk mats (Supplementary Figure 5), showing a significant reduction in cyanobacteria *Tychonema* in the water column. Ulvibacter and Citreitalea (Bacteroidota), and Xanthomonadaceae and *Pseudomonas* (Proteobacteria) showed a significant logfold increase in the water column. In the eukaryotic community, 81 taxa were significantly different between water and bulk mats (Supplementary Figure 6), showing a significant increase in several genera in the microbial mats including Bacillariophyceae (Diatoms), Vampyrellidae (Cercazoa), *Pleurastrum* (Chlorophyta), and *Obertrumia* and *Stokesia* (Ciliophora). In the water column, an increase of 13 taxa was observed including Cryptomondales (including *Geminigera*) and *Paraphysomonas*.

**Figure 5:**
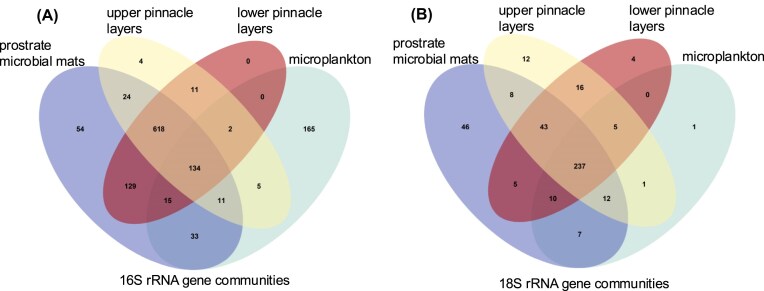
Venn diagram of shared and unique Amplicon Sequence Variants in (A) 16S and (B) 18S rRNA gene datasets of Lake Joyce. Prostrate microbial mat includes the prostrate samples (P and PD), upper pinnacle layers includes the upper layers of the dissected isolated (DU), finger (FU), and webbed (WU) pinnacles samples, lower pinnacle layers includes the lower layers of the dissected isolated (DU), finger (FU), and webbed (WU). Microplankton includes the water column samples (WTR) at all depths.

The number of shared and unique ASVs between sample types (prostrate microbial mat, water microplankton, upper layers of microbial pinnacles, lower layer of microbial pinnacles) was also grouped irrespective of sampling location and shown in Fig. 5. The water column had 165 unique bacterial and archaeal ASVs, in comparison to only one unique eukaryotic ASV in the mats (Fig. 5). The prostrate microbial mat had a significantly higher diversity than planktonic samples in two diversity indices for the 16S rRNA gene dataset (Mann–Whitney U test: Observed W = 105, *P* < 0.01 and Shannon W = 95, *P* < 0.01), and three indices for the 18S rRNA gene dataset (Mann–Whitney U test: Observed W= 108, *P* < 0.01, Shannon W= 107, *P* < 0.01, and InvSimpson W = 104, *P* < 0.01) (Fig. 4).

## Discussion

Lake Joyce is a perennially ice-covered lake and supports diverse and rich microbial communities, contributing to biomass production and biodiversity like many other lakes in the Polar regions. In our study, we provide the first comprehensive characterization of bacteria, archaea, and microbial eukaryotes using 16S rRNA and 18S rRNA gene sequencing of in both benthos and microplankton at intervals within the stratified water column for Lake Joyce during the austral summer.

### Planktonic communities across the stratified water column

The planktonic communities of Lake Joyce exhibit vertical stratification with patterns of distribution influenced by physiochemical properties of the water column. Stratified microbial communities are a key feature of meromictic lakes caused by extreme vertical chemical, redox and PAR gradients (Laybourn-Parry et al. 1995, Comeau et al. 2012, Andrei et al. 2015). As is seen in other stratified lakes in the MDV, and unsurprisingly, oxygen appears to be a key influence on the distribution of major bacterial phyla (Vick-Majors et al. 2014, Kwon et al. 2017). PAR likely played a role in structuring microbial communities with the decreasing trend in potentially phototrophic organisms with depth. At the time of sampling there was a distinct shift in relative abundance from Bacteroidetes and Cyanobacteria in the upper waters to Desulfobacterota and Patescibacteria with depth. Beyond the euphotic zone boundary (22–27 m), Candidatus *Nitrosopumilus*, an ammonia-oxidizing archaeon, reached its highest relative abundance and Firmicutes increasing within the oxic-anoxic transition zone and anoxic zone of Lake Joyce, which agreed with previously published data on bacterial distribution across several MDV lakes (Kwon et al. 2017). Desulfobacterota, formerly Deltaproteobacteria, are often sulphate reducers and likely play a role in the sulphur cycle of Lake Joyce. The increase of iron-oxidizing Proteobacteria *Gallionella* in the deep anoxic water may be linked to the increase of reduced iron with depth (Shacat et al. 2004).

For the 18S rRNA gene microbial communities, Cryptophyceae dominated the water column of Lake Joyce but experienced a negative trend in the deeper zones of the water column. This included *Geminigera cryophila*, the most abundant Cryptophyceae, which has been previously identified in nearby Lakes Bonney and Fryxell (Li and Morgan-Kiss 2019). Ochrophyta showed an opposing trend, increasing with depth in the water column. *Paraphysomonas*, a heterotrophic chrysophyte, showed highest relative abundance at the chemocline, and has previously been associated with suboxic habitats in McMurdo Dry Lakes (Li and Morgan-Kiss 2019). Additionally, an increase in mixotrophic *Nannochloropsis* with depth was observed which reached peak relative abundance at 37 m. Biotic interactions may influence the vertical distribution of mixotrophs in the water (e.g. competition between heterotrophs and mixotrophs) (Roberts and Laybourn-Parry 1999).

Several of the planktonic taxa identified, including *Nannochloropsis, Geminigera*, and *Ochromonas*, are mixotrophs (Roberts and Laybourn-Parry 1999). Mixotrophy may be a useful strategy for surviving in the extreme MDV conditions (Roberts and Laybourn-Parry 1999, Patriarche et al. 2021). Mixotrophy in extreme latitudes has been linked to PAR availability and survival in light-limited conditions, with evidence supporting that organisms switch from autotrophy to heterotrophy to survive the polar night (Thurman et al. 2012). Studies have shown that mixotrophic organisms have a higher grazing impact than heterotrophs in polar lakes (Thurman et al. 2012). This may account for their persistence into the aphotic zone of Lake Joyce at a depth of 25 m. Previous study indicates that populations of cryptophytes are negatively correlated with salinity, which in turn could restrict their distribution to the shallower waters of Lake Joyce (Li and Morgan-Kiss 2019).

### Diverse bacteria and microeukaryotes in microbial mats

The bacterial and archaeal communities of Lake Joyce were composed of several phyla, dominated by Proteobacteria and Cyanobacteria, indicating a complex community able to capitalize on the available resources of this extreme environment. The richness of microbial eukaryotes was lower than bacteria but encompassed a wide range of evolutionary lineages, which comprised both primary producers and predators in the food web.

Diatoms are often a substantial component of Antarctic microbial mats (Esposito et al. 2008, Stanish et al. 2013). In this study, the diatoms were found in the microbial mats but were largely absent for the water column, confirming a benthic niche for this group in Lake Joyce. It was not possible to identify the most abundant diatom ASVs to a higher level, highlighting a potential issue with the sensitivity of the 18S rRNA region amplified, as well as a potential underrepresentation of diatoms in the database used. However, diatoms are well documented in benthic communities in Antarctic lakes; benthic diatom flora such as species of *Craticula, Diadesmis, Navicula*, and *Nitszchia* have been identified in benthos of lakes in the MDV (Spaulding et al. 1997, , Jungblut et al. 2016), Amery Oasis (Cremer et al. 2004), Bunger Hills (Gibson et al. 2006, and Larsemann Hills, Sabbe et al. 2004).

In Lake Joyce, ciliates were highly abundant, particularly within benthic communities. Patterns of ciliate distribution observed lend support to previous studies on the importance of ciliates as potentially important predators in the truncated food webs of benthic and pelagic polar microbial communities (Xu et al. 2014).

Cyanobacteria are a fundamental component of phototrophic communities across polar freshwater and terrestrial ecosystems, playing important roles in nutrient and carbon cycling and biomass accumulation (Vincent 2000). In the microbial mats of Lake Joyce, *Tychonema, Pseudanabaena*, and *Leptolyngbya* were consistently identified in all samples of benthic mats. These findings corroborate previous assessments of Lake Joyce microbial mats through microscopy (Hawes et al. 2011, Mackey et al. 2015) and clone library surveys (Zhang et al. 2015). In previous studies by Mackey et al. (2015, 2017) abundant large oscillatorian morphotypes were identified as *Phormidium autumnale* in the mats, and it was noted that these were relatively most abundant in shallow water. In our study, the most abundant cyanobacterial genus was classified as *Tychonema* using a classifier based on the SILVA 138 database. This genus is closely related and morphologically similar to *Phormidium autumnale* (genus now revised as *Microcoleus*) both falling within a poorly resolved clade (Strunecky et al. 2013). We found the relative abundance of this ASV to decrease with depth, and it is therefore likely that the morphotypes referred by Mackey et al (2015), and this current research are the same.

Lake Joyce hosts three-dimensional pinnacles growing from prostrate microbial mats that were separately analyzed in our study. We found the upper and lower layers of the three different types of pinnacles in Lake Joyce to show differences in community composition, with a more photosynthetic community on the upper layers and heterotrophic community in the lower layers. Previous studies have found similar functional shifts within laminated microbial mats (Jungblut et al. 2016, Greco et al. 2024, Grettenberger et al. 2023).

The motile large-morphotype *Tychonema* had a higher relative abundance in the flat mat and finger pinnacle sample than in webbed and isolated pinnacles, whereas *Pseudanabaena* and *Leptolyngbya* morphotypes appear more associated with webbed and isolated pinnacles. The abundance of *Tychonema* in the finger-shaped pinnacle is consistent with its smooth surface topography even though it has significant relief above the lake floor. In this sample, the vertical relief of the pinnacle was caused by a trapped air bubble lifting the mat, which stretched upward in response to the lift (e.g. Juarez Rivera et al. 2025), whereas deeper webbed and isolated, cuspate pinnacles had no bubbles and appeared to be generated by directional growth. A high abundance of large oscillatorian cyanobacteria including *Microcoleus* (former name *Phormidium autumnale*) has been previously associated with the reduction of branching in cyanobacterial stromatolites of Lake Joyce (Mackey et al. 2018). While our findings are based on replicates from a single finger-shaped pinnacle (due to logistical constraints, it supports the hypothesis that the composition of the cyanobacterial community is related to the resulting three-dimensional structure. *Leptolyngbya*, or related genera, has been associated with vertical growth across several studies (Reyes et al. 2013, Sumner et al. 2016, Suosaari et al. 2018). *Pseudanabaena* has also been associated with textured mats and the formation of biofilms with intersecting ridges (Shepard and Sumner 2010). *Tychonema/Microcoleus*, conversely, is associated with smoothing of microbial mats consistent with the finger shaped morphology.

### Metazoa in Lake Joyce

The metazoan component of Lake Joyce was largely composed of Tardigrada, Rotifera, Arthropoda, Platyhelminthes, and sparse Nematoda. The mats had the most diverse metazoan community, while the water column was mostly populated by rotifers and arthropods, most other phyla present in mats being absent or extremely low in abundance in the water column. However, relative abundance and diversity data have to be interpreted with caution due to the lack of resolution of the 18S rRNA gene and multicellularity of metazoan and the limitations in the representation of Antarctic organisms within databases.

Lake Joyce is the only currently known MDV lake with a thriving, abundant and endemic population of an epibenthic species of copepod, identified as a new species *Diacyclops joycei* (Karanovic et al. 2014) and here likely represented by the 18S rRNA gene that was linked to copepod order Cyclopoida. Other MDV lakes have been extensively searched for crustaceans with no large population found (Roberts et al. 2004) though, a single sub-adult copepod (classified as *Boeckella* sp.) was reported by Hansson et al. (2011) from the water column of Lake Hoare. With metagenomic sequencing data from Lake Fryxell not reporting any copepods (Dillon et al. 2020a, 2020b), but with them found in samples from Lake Joyce that were collected in similar ways, we consider that our data supports the apparent exclusion of *D. joycei* from other nearby lakes and that their dispersal over even short distances is rare. Surveys of other Antarctic lakes and using environmental DNA and more specific gene markers including COI could help improve the understanding of species richness and biogeography of copepods in the McMurdo Dry Valleys and Victorialand.

### Microbial mat and microplanktonic habitat coupling in Lake Joyce

Our 16S and 18S rRNA gene microbiome survey of Lake Joyce enables a direct comparison of benthic and planktonic communities where samples are collected from similar zones within the water column. We consider that the isohaline, isothermal upper layer of the lake, extending from below the ice to 16 m depth can be considered as a single convectively mixed physical unit. Within the chemocline, conditions change rapidly with depth, convective mixing is likely absent, and direct comparisons of benthic and planktonic communities must be made with care. Within these constraints, it is clear that the composition and relative abundance of both 16S and 18S rRNA genes were distinct between the microbial mat and water habitats in Lake Joyce, mirroring findings from other MDV sites at Lake Vanda (Ramoneda et al. 2021). The water column was significantly less diverse in prokaryotes and microbial eukaryotes than the benthic communities. This suggests, unsurprisingly, that differences in the communities that assemble in the benthos and plankton are commonplace in Antarctic Dry Valley lakes, with communities occupying different environmental niches provided by these distinct systems. However, a considerable number ASVs were shared between the habitats and suggested a level of connectivity between the habitats that could potentially support cross flow of energy and biological matter.

The presence of copepods in Lake Joyce perhaps sets them apart in from other lakes in terms of benthic-pelagic coupling. In marine environments, copepods can perform ontogenetical migration across the seasons in marine environments which has been associated with downward carbon fluxes including the subarctic Pacific Ocean. It therefore raises the possibility of *D. joycei* playing a role in the connectivity of carbon and microbial diversity in benthic and water column habitats in Lake Joyce as well. The occurrence of shared ASVs between benthic and planktonic habitats may be indicative of interchange of organisms, though it can also be due to methodological contamination or passive deposition.

We found that the most ASVs found in the plankton samples in the mixed upper layers of the water column were also found in the comparable benthic samples, while the benthos contained many that were not so shared. This perhaps suggests that the simple explanation of the shared ASV requires a mechanism that delivers plankton to the benthos rather than vice-versa, and hence a simple sedimentation process may be most likely.

The proportion of shared 16S rRNA gene taxa was the mats and planktonic communities, and the water had more unique bacteria and archaea whereas microbial mats were richer in microbial eukaryotes. There was only one unique 18S rRNA gene ASV for the microplankton. Key drivers of these compositional changes were filamentous cyanobacteria in particular the highly abundant mat former *Tychonema* and diatoms in the benthos and Cryptophytacea in the water column. This suggests that microbial mats with higher biomass stocks are likely enriched nutrient concentrations due retention of nutrients and recycling (Bonilla et al. 2005, Varin et al. 2010). While the water column has more stringent filtering of microbial communities along the physicochemical gradients and oligotrophic water conditions. The findings also imply that certain bacteria and archaea are predominantly or exclusively present in the water column, whereas the microbial eukaryotes that dominate the water column are also present in lower abundances in the benthic environment. Few studies have simultaneously studied the benthic and planktonic communities of polar lakes; therefore this study offers new insights into the connectivity and coupling of these environments. The study is restricted to sample collection during the austral summer and it would be highly valuable to extend sampling across seasons especially winter conditions with 24 h darkness. However our study still highlights the need to consider both the planktonic and benthic communities to study biodiversity and productivity of ice-covered lakes in Antarctica.

A limitation of the methods used in this study is that DNA-based methods amplify relic DNA in the biofilms and water column. This may be augmented by the cold, alkaline conditions of Lake Joyce that potentially preserve DNA fragments over long time scales (Carini et al. 2016), especially for the lower microbial mat samples. However, there is a good consensus in cyanobacterial communities with the previously identified morphotypes (Mackey et al. 2015) and other DNA-based taxonomic studies of microbial communities in McMurdo Dry Valley Lakes (Jungblut et al. 2016, Grettenberger et al. 2023), suggesting that the cyanobacterial portion of the mats were adequately characterized. Furthermore, a previous study suggested that relict DNA has a limited influence on estimates of taxonomic and phylogenetic diversity (Lennon et al. 2018). Future studies could identify relic DNA by treating samples using a propidium monoazide protocol or employing RNA sequencing to circumvent the issue of legacy DNA. Amplicon studies may also have some inherent biases, such as PCR bias from the selected universal primers. The 18S rRNA primers used in this study have documented coverage gaps (Kounosu et al. 2019), in particular for Fungi meaning that the diversity and contribution of these groups may have been underestimated in this dataset. PCR independent methods, such as metagenomics, reduce certain methodological biases in addition to allowing untapped parts of the communities (e.g. viruses) to be explored.

## Conclusions

The study represents the first comprehensive 16S and 18S rRNA gene sequencing study of the microbial communities in the benthic and planktonic habitats of perennially ice-covered Lake Joyce in Antarctica during the austral summer. Comparison of bacteria, archaea, and microbial eukaryotes forming the microbial mats and microplankton showed that the communities vary in their community composition, though with some taxa shared between both communities. Overall, the laminated benthic mats supported more biological richness than the plankton, and were particularly important in terms of supporting eukaryotes and metazoa; highlighting the importance of microbial mats for supporting biodiversity. The results also suggest that microbial richness and community composition in Lake Joyce is shaped by an intricate interplay between multiple environmental conditions including at least dissolved oxygen, PAR and sedimentation. Understanding the environmental drivers of microbial community assemblages in Antarctic lakes will help to better predict the impact of future climatic-driven environmental change.

## Supplementary Material

fiag078_Supplemental_File
