# Supplementary material for: Distinct benthic and planktonic microbial communities within the stratified water column of the perennially ice-covered Lake Joyce, Antarctica
Source: FEMS Microbiol Ecol. 2026 Aug 6;102(8):fiag078. doi: 10.1093/femsec/fiag078 (PMC13452585; doi:10.1093/femsec/fiag078)
Supplement: Supplementary file 1 [file fiag078_supplemental_file.docx]

**Supplementary Material**

**Distinct benthic and planktonic microbial communities within the stratified water column of the perennially ice-covered Lake Joyce, Antarctica**

**Supplementary Table S1:** Highest cultured and uncultured BLAST match of the 19 most abundant Cyanobacteria ASVs identified in Lake Joyce, Antarctica.

| dad2_name | ASV_name | cultured BLAST match | Accession number | % | Uncultured BLAST match |  | Accession number | % |
| --- | --- | --- | --- | --- | --- | --- | --- | --- |
| 2629ac2b78514355aa0f84f2744fb028 | ASV1 | Pseudanabaena_PCC-7429 | MT298092.1 | 100 | Uncultured cyanobacterium clone LV30_213 | | KM112137.1 | 100 |
| 00f20d641a58e3c82c5756568ad89548 | ASV2 | Pseudanabaena_PCC-7429 | LT600733.1 | 100 | Uncultured bacterium clone Otu259 | | MH728254.1 | 100 |
| cc7083382d1b6af8feb121cfe2779359 | ASV3 | Phormidium_CYN64 | JQ687330.1 | 100 | Uncultured cyanobacterium clone LV30_168 | | KM112159.1 | 100 |
| be19b946080a75ade3e89d32c052b53e | ASV4 | Phormidium_CYN64 | MK211231.1 | 98.81 | Uncultured cyanobacterium clone LV20_387 | | KM112151.1 | 100 |
| 1eb4ef4c3db9039356f514a629cf91a3 | ASV5 | Vampirovibrionales | NR_104911.1 | 91.67 | Uncultured bacterium clone 2695 | | KX704889.1 | 99.6 |
| 89e057f522bef6d66e3ee8a398653a34 | ASV6 | Vampirovibrionales | KY778750.1 | 85.7 | Uncultured bacterium clone 4140 | | KX706444.1 | 99.21 |
| d3b66e47d5ab86a627838df50294ed61 | ASV7 | Vampirovibrionales | MK861887.1 | 87 | Uncultured bacterium clone js6204 | | KY256697.1 | 94.07 |
| 47153dd89d148375215aaaca9717b450 | ASV8 | Obscuribacteraceae | MN072766.1 | 91.7 | Uncultured bacterium clone OTU_7551 | | KU227968.1 | 99.6 |
| 819f9e1c1b3bcc525a11d73d6e6442cf | ASV9 | Synechococcus_PCC-7502 | AB075995.1 | 94.88 | Uncultured cyanobacterium clone LV30_196 | | KM112156.1 | 100 |
| 89e0a914f573238e3d70c9d9303e8aac | ASV10 | Synechococcus_PCC-7502 | AB075995.1 | 95.28 | Uncultured cyanobacterium clone LV30_196 | | KM112156.1 | 99.6 |
| 7fb5a36541cfcf1a186efa5a8f707d67 | ASV11 | Leptolyngbyaceae | KU219729.1 | 100 | Uncultured cyanobacterium clone LH8_90 | | KM112104.1 | 100 |
| d5bf72725229f9ad810707b6c8e0c9b6 | ASV12 | Leptolyngbya_ANT.L67.1 | MN243119.1 | 100 | Uncultured cyanobacterium clone LH21_422 | | KM112128.1 | 100 |
| 27993c9c3f153e8f4b2b92956d4086f7 | ASV13 | Leptolyngbyaceae LB3-76 | KM052844.1 | 100 | Uncultured cyanobacterium clone LV30_172 | | KM112161.1 | 100 |
| 79aeef6159351463c3618c62dbfa39ac | ASV14 | Chamaesiphon_PCC-7430 | JQ687333.1 | 99.21 | Uncultured cyanobacterium clone LJ6_347 | | KM112131.1 | 100 |
| 563dafa7ecbdaec2addabcff3bdce142 | ASV15 | Chamaesiphon_PCC-7430 | JQ687333.2 | 99.6 | Uncultured cyanobacterium clone LH21_463 | | KM112129.1 | 100 |
| 9fde9971ce2276d0b46da55cd7aafa0a | ASV16 | Chamaesiphon_PCC-7430 | MG710502.1 | 97.23 | Uncultured cyanobacterium clone LH16_275 | | KM112120.1 | 100 |
| 349e47f1a490702643a56067bf13d587 | ASV17 | Geitlerinema_LD9 | MT488241.1 | 100 | Uncultured cyanobacterium clone Ant28_cl23 | | KM052826.1 | 100 |
| 028bf3336d8421616d1c0429f0c3feb5 | ASV18 | Geitlerinema_LD9 | KT315939.1 | 99.21 | Uncultured bacterium clone OTU_62 | | KU221559.1 | 99.6 |
| 4c327505a6047f3d55fb7f6a74f1a0a8 | ASV19 | Tychonema_CCAP_1459-11B | MT013207.1 | 100 | uncultured bacterium partial 16S rRNA gene | | LR595009.1 | 100 |

**Supplementary Table S2:** Differentially abundant bacterial taxa in pinnacle structures calculated using DESeq2

| **Taxon** | **log2FoldChange** | **adjusted_p_value** |
| --- | --- | --- |
| S-BQ2-57_soil_group | 3.82 | 0 |
| Luteolibacter | -5.21 | 0 |
| Rhodanobacteraceae | 1.87 | 0 |
| Microbacteriaceae | -3.12 | 0 |
| Pseudoxanthomonas | -6.49 | 0 |
| TRA3-20 | 9.89 | 0 |
| Sphingorhabdus | -5.63 | 0 |
| Sphingopyxis | -4.01 | 0 |
| SM1A02 | 1.89 | 0 |
| Cryobacterium | -2.5 | 0 |
| Polaromonas | -1.42 | 0 |
| Parcubacteria | -2.03 | 0 |
| Leptolyngbya | 3.42 | 0 |
| Pirellulaceae | -3.27 | 0 |
| Ahniella | 9.13 | 0 |
| Beijerinckiaceae | 2.56 | 0 |
| Ilumatobacter | -2.41 | 0 |
| Caldilineaceae | 1.33 | 0 |
| Gammaproteobacteria_Incertae_Sedis | -8.86 | 0 |
| Altererythrobacter | -7.31 | 0 |
| Oligoflexus | -2.19 | 0 |
| Polyangia | -8.35 | 0 |
| JGI_0001001-H03 | -3.21 | 0 |
| oc32 | 7.97 | 0 |
| Terrimicrobium | -1.33 | 0 |
| Candidatus_Berkiella | -2.56 | 0 |
| Methylotenera | -2.83 | 0 |
| Truepera | 4.1 | 0 |
| CCM19a | -1.23 | 0 |
| GOUTA6 | 7.46 | 0 |
| SC-I-84 | 2.23 | 0 |
| Babeliales | -2.27 | 0 |
| Porphyrobacter | -7.97 | 0 |
| OLB12 | 3.07 | 0 |
| Candidatus_Pacebacteria | 7.67 | 0 |
| Roseimaritima | -4.72 | 0 |
| Roseomonas | 2.75 | 0 |
| Sphingobacteriaceae | -7.91 | 0 |
| Symbiobacteraceae | 7.37 | 0 |
| Vermiphilaceae | -2.72 | 0 |
| Actinomarinales | 2.3 | 0 |
| OM27_clade | 5.22 | 0 |
| CM1G08 | 7.25 | 0 |
| Hydrogenophaga | -4.97 | 0 |
| Ga0077536 | 1.7 | 0 |
| PB19 | 2.65 | 0 |
| Burkholderiales | 2.25 | 0 |
| Oligoflexales | -5.77 | 0 |
| Pirellula | -1.91 | 0 |
| Pseudohongiella | 4.23 | 0 |
| UBA12409 | -4.61 | 0 |
| BIrii41 | 7.15 | 0 |
| Sphingomonas | -1.45 | 0 |
| Paenibacillaceae | 7.08 | 0 |
| Micropepsaceae | -5.74 | 0 |
| Bradymonadales | 6.93 | 0 |
| Myxococcaceae | 6.88 | 0 |
| Aquicella | -2.67 | 0 |
| Xanthomonadales | -1.69 | 0 |
| KD3-93 | 6.82 | 0 |
| Legionella | -2.39 | 0 |
| Rickettsiaceae | -5.64 | 0 |
| NS9_marine_group | 2.68 | 0 |
| Lysobacter | -5.35 | 0 |
| Sumerlaea | 6.9 | 0 |
| CL500-3 | 4.04 | 0 |
| WD2101_soil_group | -1.88 | 0 |
| Rhodovastum | 2.6 | 0 |
| WWE3 | 4.92 | 0 |
| P3OB-42 | 2.44 | 0 |
| Vampirovibrionales | 2.99 | 0 |
| Novosphingobium | -1.55 | 0 |
| Gammaproteobacteria_Incertae_Sedis | 2.37 | 0 |
| Sediminibacterium | 2.13 | 0 |
| JG30-KF-CM45 | 1.8 | 0 |
| Rhizobiales_Incertae_Sedis | 1.96 | 0 |
| 67-14 | -2.07 | 0 |
| Rhodobacteraceae | -1.27 | 0 |
| Absconditabacteriales_(SR1) | -1.68 | 0 |
| MND1 | 1.51 | 0 |
| Labrys | 6.26 | 0 |
| 37-13 | 6.19 | 0 |
| Gallionellaceae | 6.45 | 0 |
| Silvanigrellaceae | 2.43 | 0 |
| OM60(NOR5)_clade | -1.21 | 0 |
| Gemmatimonas | -1.3 | 0 |
| Candidatus_Xiphinematobacter | -1.47 | 0 |
| Acidimicrobiia | 2.07 | 0 |
| Nitrosomonadaceae | 4.31 | 0 |
| KI89A_clade | 3.1 | 0 |
| Saccharimonadales | 1.68 | 0 |
| mle1-27 | 1.81 | 0 |
| Chiayiivirga | -2.24 | 0 |
| Sporichthyaceae | 1.06 | 0 |
| Ferruginibacter | -2.79 | 0 |
| Holosporaceae | -1.66 | 0 |
| Blastocatellaceae | -1.35 | 0 |
| Pseudorhodobacter | -1.93 | 0 |
| Nitrosomonas | 1.66 | 0 |
| KD4-96 | 1.28 | 0 |
| Sva0996_marine_group | -1.29 | 0 |
| SH3-11 | 2.22 | 0 |
| Oikopleura | 3.53 | 0 |
| Burkholderiaceae | 1.17 | 0 |
| cvE6 | -3.53 | 0 |
| Neochlamydia | -2.1 | 0 |
| Actinobacteria | -1.95 | 0 |
| Phenylobacterium | -3.64 | 0 |
| Gracilibacteria | -3.54 | 0 |
| Verrucomicrobiaceae | -1.24 | 0 |
| Gaiella | 1.28 | 0 |
| Alphaproteobacteria | -1.54 | 0 |
| Ellin6067 | 1.02 | 0 |
| SM1A07 | 5.46 | 0 |
| Opitutaceae | 3.41 | 0 |
| Vicinamibacteraceae | -0.78 | 0 |
| Aureispira | 5.59 | 0 |
| Rhodopirellula | -1.66 | 0 |
| Chitinophagales | 3.9 | 0 |
| Defluviicoccales | 5.36 | 0 |
| Microtrichaceae | -1.25 | 0 |
| Psychroglaciecola | -1.6 | 0 |
| Methylacidiphilaceae | -3.47 | 0 |
| Algoriphagus | -1.54 | 0 |
| Fimbriiglobus | -1.5 | 0 |
| Candidatus_Peregrinibacteria | 5.25 | 0.01 |
| Parachlamydiaceae | -2.77 | 0.01 |
| Hyphomonas | -1.35 | 0.01 |
| DEV007 | -1.64 | 0.01 |
| Sandaracinobacter | -6.73 | 0.01 |

**Supplementary Figure 1:** Temperature, pH, dissolved oxygen, specific conductivity, chlorophyll-*a*, and phycocyanin water column profile of Lake Joyce, measured in November , 2014

**Supplementary Figure 2 -** Distribution of four major spectral algal classes (green algae, cyanobacteria, mixed and cryptophytes) were determined in Lake Joyce using *in situ* Chl-*a* fluorescence measurement with a submersible spectral fluorometer (FluoroProbe, BBE Moldaenke).

**Supplementary Figure S3 -** Evolutionary placement analysis of Cyanobacterial Amplicon Sequence variants (ASVs) from Lake Joyce. ASVs are numbered in order and their SILVA assignment can be found in Table S2.

**Supplementary Figure S4** - Hierarchical clustering of dissected pinnacles for **A** 16S rRNA gene and **C** 18S rRNA gene ASVs. Alpha diversity indices Observed, Shannon, and InvSimpson for **B** 16S rRNA and **D** 18S rRNA dissected structure datasets. Abbreviations: FU, upper layer of finger shaped pinnacle, FL, lower layer of finger shaped pinnacle, DU upper layer of isolated pinnacle; DL, lower layer of isolated pinnacle; WU, upper layer of webbed pinnacles; WL, lower layer of webbed pinnacle.

**Supplementary Figure S5 -** Differentially abundant bacterial taxa significant to p < 0.01. The 100 most abundant taxa are shown at genus level or highest taxonomic assignment. Positive log2fold change indicated increase in the water column and negative log2fold change indicates increase in the bulk flat microbial mat communities. Relative abundance (%) is shown in a heatmap. Abbreviations: WTR, water column P, prostrate mat; PD, prostrate mat nearer to delta; FU, upper layer of finger shaped pinnacle, FL, lower layer of finger shaped pinnacle, DU upper layer of isolated pinnacle; DL, lower layer of isolated pinnacle; WU, upper layer of webbed pinnacles; WL, lower layer of webbed pinnacle.

**Supplementary Figure S6** - Differentially abundant eukaryotic taxa significant to p < 0.01. Taxa are shown at genus level or highest taxonomic assignment. Positive log2fold change indicated increase in the water column and negative log2fold change indicates increase in the bulk flat microbial mat communities. Relative abundance (%) is shown in a heatmap. Abbreviations: WTR, water column; P, prostrate mat; PD, prostrate mat nearer to delta; FU, upper layer of finger shaped pinnacle, FL, lower layer of finger shaped pinnacle, DU upper layer of isolated pinnacle; DL, lower layer of isolated pinnacle; WU, upper layer of webbed pinnacles; WL, lower layer of webbed pinnacle.
